# A retrospective study of shrinking field radiation therapy during chemoradiotherapy in stage III non-small cell lung cancer

**DOI:** 10.18632/oncotarget.23849

**Published:** 2018-01-03

**Authors:** Chenxue Jiang, Shuiyun Han, Wucheng Chen, Xiaozhen Ying, He Wu, Yaoyao Zhu, Guodong Shi, Xiaojiang Sun, Yaping Xu

**Affiliations:** ^1^ First Clinical Medical School, Wenzhou Medical University, Wenzhou, PR China; ^2^ Department of Radiation Oncology, Zhejiang Cancer Hospital, Hangzhou, PR China

**Keywords:** lung cancer, chemoradiation therapy, dose escalation, adaptive radiotherapy

## Abstract

**Background:**

and purpose: This retrospective study aimed to investigate the feasibility of shrinking field radiotherapy during chemoradiotherapy in non-small cell lung cancer (NSCLC).

**Patients and methods:**

Ninety-seven patients with stage III NSCLC who achieved a good response to chemoradiation were analyzed. Computed tomography was performed after 40-50 Gy dose radiation to evaluate curative effect. Patients in the shrinking field group underwent resimulation CT scans and shrinking field radiotherapy. Acute symptomatic irradiation-induced pneumonia (ASIP), progression patterns and survival were assessed.

**Results:**

Of the 97 patients who achieved response after a median total dose of 60 Gy, fifty patients received shrinking field radiotherapy. The incidence of acute symptomatic irradiation-induced pneumonia tended to be lower for the shrinking field group (18.0% vs. 23.4%, *P =* 0.51). The rate of disease progression was significantly higher in the non-shrinking than shrinking field group (95.7% vs. 66.0%, *P* < 0.001). Compared to the non-shrinking field group, the shrinking field group had similar overall survival (30.0 vs. 30.0 months, *P =* 0.58) but significantly better median progression-free survival (14.0 vs. 11.0 months, *P =* 0.006).

**Conclusions:**

Shrinking field radiotherapy during chemoradiotherapy in stage III non-small cell lung cancer seems safe with acceptable toxicities and relapse, and potentially spares normal tissues and enables dose escalation. Prospective trials are warranted.

## INTRODUCTION

A multidisciplinary approach is the standard treatment for locally advanced non-small cell lung cancer (NSCLC), especially for unresectable stage IIIA and IIIB disease. Research in NSCLC has shown that with each additional Gy of radiation, long-term (3- to 5-year) locoregional tumor control improves by 1% and the risk of death reduces by 3% [1]. Modern technology such as three-dimensional conformal treatment (3D-CRT) and intensity-modulated radiation therapy (IMRT) have enabled improvements in the conformity of dose distributions [2, 3]. However, radiation-induced lung injury (RILI), a dose-limiting complication, remains an obstacle to radiation dose escalation [4, 5] and negatively affects patient quality of life.

Tumor regression has been observed in NSCLC in clinical practice and reported during the course of radiotherapy [6–8]. Guckenberger et al. reported continuous tumor regression of 1.2% per day during simultaneous chemoradiation and a reduction in the consequent residual gross tumor volume (GTV) of 49 ± 15% after six weeks of treatment [9]. Thus shrinkage of the target volume during radiation therapy seems appropriate to enable delivery of a higher dose to the target and better sparing of normal tissues. A previous study by Nkhali et al. [10] recalculated the radiation plan for target volume reduction based on a 18F-deoxyglucose positron emission tomography /computer tomography (FDG-PET/CT) scan on day 21 of chemoradiotherapy, and found replanning increased the total dose from 50 to 66 Gy with the largest absolute benefits for the lung V20 (percentage lung volume irradiated at doses exceeding 20 Gy) [median –2.15% (range –5.4–0.2%)] and heart V40 [median –1.8% (range –7.1–2.2%)].

Efforts towards facilitating higher dose escalation by assessing tumor shrinkage during treatment have been made in NSCLC [9, 11–15]; these studies reported the feasibility of adaptive therapy during radiation in terms of superior planning and tumor control [9, 12, 14–16]; however, Gillham et al. [11] reached the opposite conclusions. Besides achieving controversial results, all of these studies evaluated patients who received replanned radiation therapy but had small sample sizes and did not select patients based on tumor response at mid-radiation therapy, such as stable disease, partial response (PR) and complete response (CR). To the best of our knowledge, the present study is the first to describe the pulmonary toxicities, failure patterns and outcomes for patients with NSCLC who have a good response to chemoradiotherapy at the second simulation CT scan during treatment.

In present work, we retrospectively reviewed 97 cases of stage III NSCLC to evaluate the feasibility of shrinking field radiotherapy after complete or partial response during chemoradiotherapy. We compared the toxicity, efficacy and survival outcomes of patients whose treatment strategy was replanned with those who received non-adapted treatment.

## RESULTS

### Patient and tumor characteristics

A total of 97 patients with NSCLC who were good responders to chemoradiotherapy (without surgery) were analyzed between September 2009 and November 2014 at our institute. Fifty patients were in the shrinking field group and 47 patients in the non-shrinking field group. All patients were of Han Chinese (East Asian) ethnicity.

Table 1 shows the characteristics of the patients in both groups. Gender, age, smoking history, histopathology, tumor location, location types and clinical stage were not significantly different between groups. The mean primary GTV and PTV values were higher for the shrinking field group than the non-shrinking field group [GTV, 116.8 cm^3^ (95% CI, 91.8–141.8 cm^3^) vs. 102.9 cm^3^ (95% CI, 76.4–129.3 cm^3^), *P =* 0.44; PTV, 493.0 cm^3^ (95% CI, 447.4–538.6 cm^3^) vs. 458.0 cm^3^ (95% CI, 405.6–510.5 cm^3^), *P =* 0.31]; see Table 1.

**Table 1:** Patient, tumor and treatment characteristics

Characteristic	Non-shrinking field group (*N* = 47)	Shrinking field group (*N* = 50)		*P* value
Gender				
Male	44 (94)	47 (94)		> 0.99
Female	3 (6)	3 (6)		
Age (yr), median (range)	57 (40–69)	57 (33-76)		0.25
Smoking history (pack-years)				
≥ 30	36 (77)	45 (90)		0.19
< 30	11 (23)	5 (12)		
Histopathology				
Squamous	32 (68)	40 (80)		0.39
Adenocarcinoma	8 (17)	6 (12)		
No specific type	7 (15)	4 (8)		
Upper lobe vs. other location				
Upper	32 (68)	32 (64)		0.67
other	15 (32)	18 (36)		
Location type				
Central type	31 (66)	35 (70)		0.67
Peripheral type	16 (34)	15 (30)		
Volume of GTV (cm3), mean, (95% CI)	102.9 (76.4–129.3)	116.8 (91.8-141.8)		0.44
Volume of PTV (cm3), mean, (95% CI)	458.0 (405.6–510.5)	493.0 (447.4–538.6)		0.31
Clinical stage				
IIIA	22 (47)	20 (40)		0.50
IIIB	25 (53)	30 (60)		
Median decreases in PTV (cm3), (range)		184.2 (28.1– 449.7)		
Dose to PTV (Gy), median (range)	60 (46-69)	60 (46–70)	0.17	
≥ 60 Gy	29 (62)	35 (70)	0.39	
< 60 Gy	18 (38)	15 (30)		
Chemotherapy				
Platinum + taxane	30 (64)	37 (74)	0.36	
Platinum +pemetrexed	5 (11)	6 (12)		
Other regimens	12 (26)	7 (14)		
Chemotherapy cycles				
≥ 4	39 (83)	37 (74)	0.28	
< 4	8 (17)	13 (26)		
Concurrent chemoradiotherapy				
Yes	28 (60)	35 (70)	0.58	
No	19 (40)	15 (30)		
Technology				
IMRT	38 (81)	43 (86)	0.49	
3D-CRT	9 (19)	7 (14)		
Dosimetric data				
V5 (%), mean, (95% CI)	48.3 (45.2–51.3)	49.7 (46.2–53.2)	0.54	
V20 (%), mean, (95% CI)	26.5 (25.0–28.0)	26.9 (25.0–28.8)	0.77	

GTV - gross tumor volume; CI - confidence interval; PTV - planned target volume. IMRT - intensity-modulated radiation therapy; 3D-CRT - three-dimensional conformal treatment; Vdose - the percentage of lung volume irradiated to doses exceeding a threshold. Platin including carboplatin, cisplatin or nedaplatin.

### Treatment

All patients received IMRT and responded to chemoradiotherapy during treatment (median total dose, 60 Gy; range, 50–70 Gy). For the adaptive plan, the target volumes were adapted on the basis of the pretreatment GTV and spared lymph node regions of presumed microscopic involvement, resulting in shrinkage of the PTV. Tumor shrinkage was assessed as the change in volume of the PTV between the original plan and the last plan using Pinnacle treatment planning system software. The median reduction in PTV between plans was 184.2 cm^3^ (range, 28.1–449.7 cm^3^) and the mean reduction in the PTV was 38.6% (95% CI, 33.7–43.4%). The dose to the PTV tended to be higher for the shrinking field group (*P =* 0.17, Mann-Whitney test), suggesting that shrinking field radiotherapy may have potential for radiation dose escalations. The total dose of 60 Gy or greater (≥ 60 Gy) rate was 70.0% and 61.7% for the shrinking field and non-shrinking field groups, respectively (*P =* 0.39). Dosimetric parameters including the V5 and V20 were similar between groups (Table 1).

Of the entire cohort, 76% of patients received at least four cycles of chemotherapy as first-line treatment. The remaining patients underwent cycle reductions due to intolerable toxicities or the patient’s choice. The chemotherapy regimens are shown in Table 1. Platinum-based therapy was used, including carboplatin and cisplatin. Univariate analysis indicated that the number of chemotherapy cycles, chemotherapy regimens, and use of concurrent chemoradiotherapy and radiotherapy technology were not significantly different between groups.

### Systematic irradiation-induced pneumonia (ASIP) and survival outcomes

The median follow-up time was 41.0 months (range, 7.0–74.0 months). The incidence of ASIP was 20.6% (20/97) for the entire cohort, 18.0% (9/50) for the shrinking field group and 23.4% (11/47) for the non-shrinking field group. The χ^2^ test indicated that shrinking field reduced pulmonary toxicity, though not significantly (*P =* 0.51) (Table 2). Grade 4 or higher toxicities were not observed in all patients.

**Table 2:** Grading of the two groups evaluated for irradiation induced lung injury

Grade	Non-shrinking field group (*N* = 47)	Shrinking field group (*N* = 50)	*P* value
Grade 0	5	5	
Grade 1	31	35	
Grade 2	9	7	
Grade 3	2	2	
≥ 2 Grade (%)	11 (23.4)	9 (18.0)	0.51

**Figure 1:**
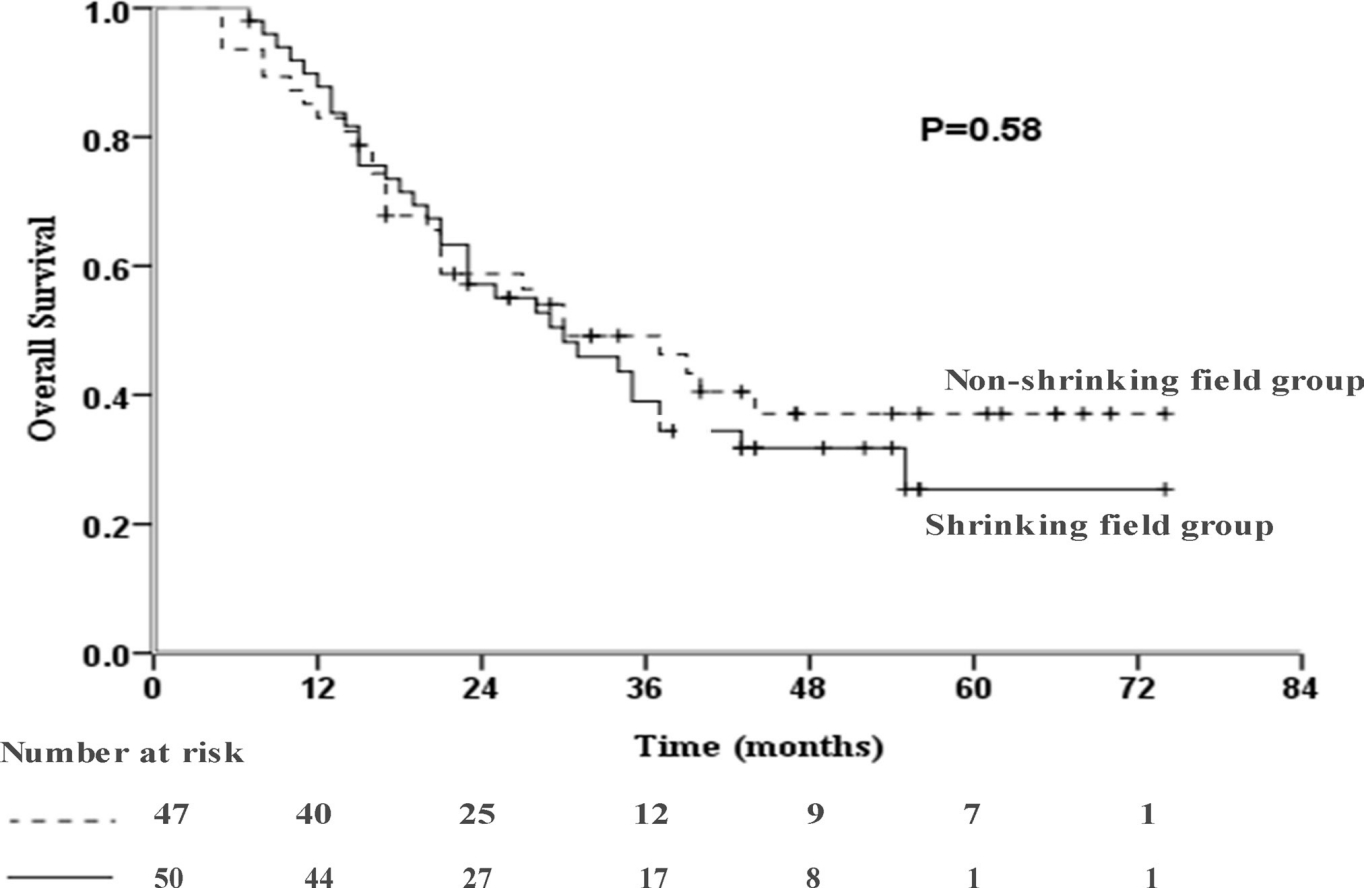
Kaplan-Meier curve of overall survival (OS) in the studied population (*P* = 0.58)

**Figure 2:**
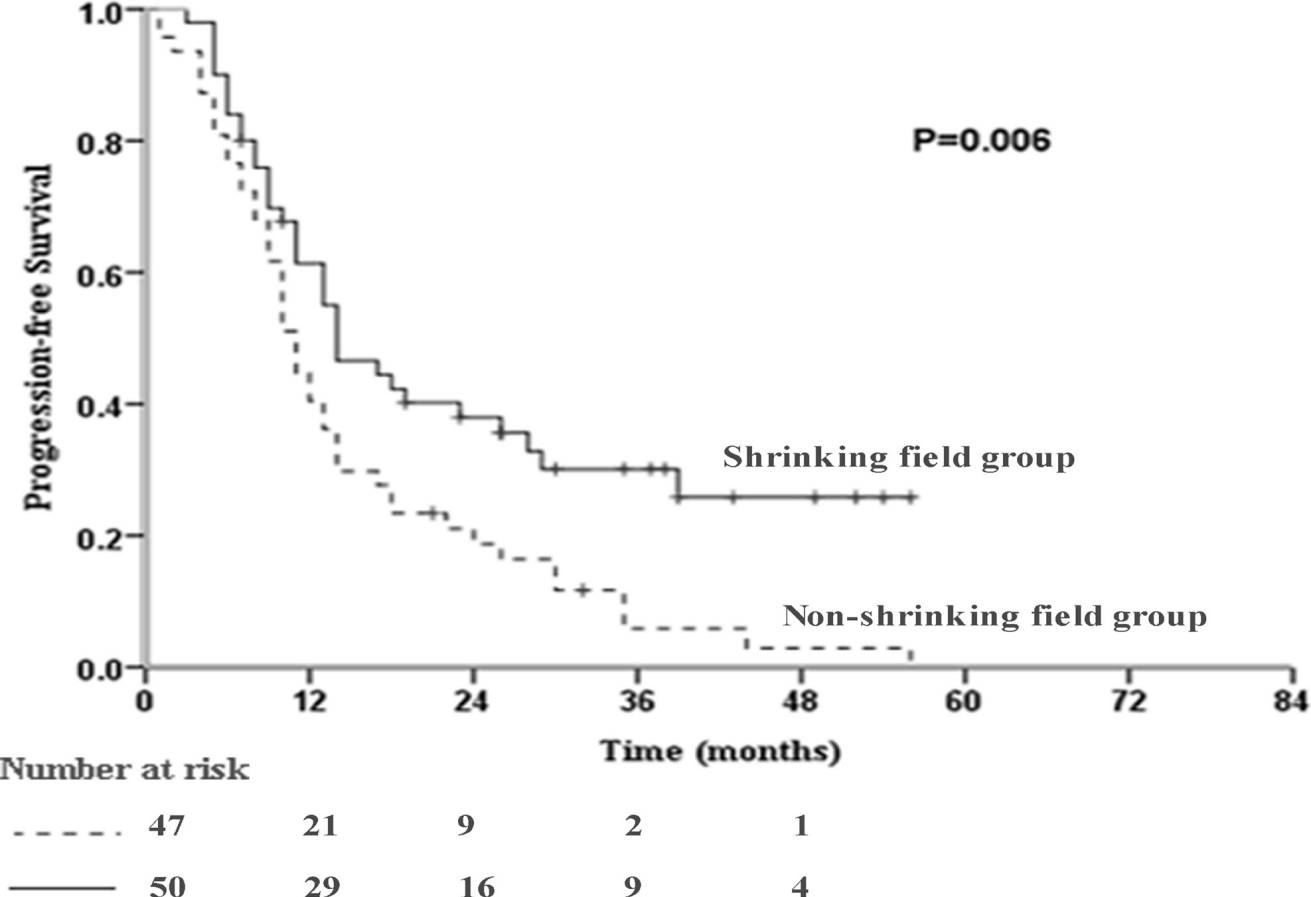
Kaplan-Meier curve of progression-free survival (PFS) in the studied population (*P* = 0.006)

The incidence of disease progression was significantly higher in the non-shrinking field group than the shrinking field group (95.7% vs. 66.0%, *P <* 0.001; Table 3). Sixteen, 13 and 4 patients in the shrinking field group and 24, 17 and 4 patients in the non-shrinking field group suffered locoregional, distant and both locoregional and distant progression, respectively. Among patients with progression, the incidence of total locoregional progression was not significantly different between the shrinking field and non-shrinking field groups (62.2% vs. 60.6%, *P =* 0.89). The impact of shrinking field on the pattern of progression was similar to that of an unadjusted radiation volume. Compared to the non-shrinking field group, the shrinking field group had a similar median OS (30.0 months, 95% CI: 18.8-41.2 months vs. 30.0 months, 95% CI: 16.5–43.5 months, *P =* 0.58; Figure 1), but significantly improved median PFS (14.0 months, 95% CI: 8.7-19.3 months vs. 11.0 months, 95% CI: 9.3–12.7 months, *P =* 0.006; Figure 2).

**Table 3:** Progression data

Relapse	Non-shrinking Field group (*n =* 47)	Shrinking Field group (*n =* 50)	*P* value
Locoregional relapse (LR)	24	16	
Distant metastasis (DM)	17	13	
LR+DM	4	4	
Total	45 (95.7%)	33 (66.0%)	*P* < 0.001

## DISCUSSION

An increased dose of radiation is associated with improved tumor control in patients with NSCLC [1, 17], but dose escalations are normally limited by the dose-volume constraints for the normal tissues, especially the lung [4]. In this study, we compared the pulmonary toxicities, failure patterns and survival outcomes for shrinking field and non-adaptive treatment in patients with NSCLC who responded (PR or CR) at mid-radiotherapy. The results indicated that patients who underwent shrinking field radiotherapy had larger tumors and received a higher dose of radiation, yet had similar dosimetric parameters to the non-shrinking field group. The incidence of progression was significantly lower in the shrinking field group. Furthermore, the shrinking field group achieved better median PFS and had a lower incidence of ASIP, but similar median OS to the non-shrinking field group. These findings suggest that field reduction for tumor shrinkage during chemoradiotherapy is a superior treatment for good responders with locally advanced NSCLC, which may prompt future dose-escalation studies.

The incidence of ASIP was higher in the non-shrinking field group, though this difference was not significant (23.4% vs. 18.0%, *P =* 0.51). Dosimetric parameters, including the V5 and V20 were similar between groups (Table 1), despite the fact the shrinking field group had a larger initial tumor volume. Thus, our findings lead us to recommend that adaptive planning has the potential to reduce the dose received by the normal tissues. Shi et al. [18] reported a patient with synchronous bilateral T2 NSCLC who received 3D conformal proton radiotherapy in combination with carboplatin/paclitaxel chemotherapy, whose tumor had shrunk by > 50% by the end of treatment. Verification plans showed that the pulmonary V20 cobalt gray equivalent (CGE) would have increased significantly from 25.3% to 31.1% (123% of the initial value) without replanning. A similar result was reported by Guckenberger et al. [9], in which the lung doses were significantly decreased by adaptive planning without compromising GTV coverage, compared to the initial plans. The single adaptation plan at week 3 or week 5, and the double adaptation plan at week 3 and week 5 reduced the MLD by 5.0% ± 4.4%, 5.6% ± 2.9% and 7.9% ± 4.8%, respectively [9]. Besides the dosimetric benefits offered to the lung tissues, shrinking field techniques also enable more patients to receive radiation by preventing the dose tolerances of the esophagus and spinal cord being exceeded [12, 15, 18]. On the basis of increased normal tissue sparing, the risk of radiation-induced toxicity may be reduced. The incidence of ASIP was not significantly different between groups in this study. We speculate that the advantages in terms of reduced toxic side effects in the shrinking field group may be more obvious if a larger cohort of patients is assessed.

The Radiation Therapy Oncology Group (RTOG) phase III clinical trial RTOG 0617 [19] showed the standard-dose (60 Gy) was better than the high-dose (74 Gy) in term of OS and treatment-related deaths. The causes leading to the unsatisfactory outcomes in the high-dose group may be the increased difficulty of patients completing concurrent chemotherapy and radiation therapy planning without adaptive therapy. As an added benefit, the shrunken radiation field could reduce compromises in target coverage during radiation planning. The internal CTV coverage of two patients in the study by Koay et al. [15] increased from 82% and 57%, respectively, to 100% for both patients after resimulation and replanning. Many studies have engaged an adaptive target strategy for radiation dose escalations [11, 20]. Gillham et al. [11] reported the GTV dose increased by 6.8 Gy in the context of lung sparing when using twice adapted radiation treatment. Two patients received a dose of 82 Gy without exceeding any normal tissue tolerances in a study by Weiss et al. [20] In the present study, more patients in the shrinking field than non-shrinking field group received a radiation dose of 60.0 Gy or greater (70.0% vs. 61.7%, *P =* 0.39). These results indicate that shrinking field is a promising methodology that enables dose escalation. However, a previous study [11], which aimed to facilitate dose escalation though reducing the target volume during radiotherapy in patients assessed using FDG PET/CT, failed to achieve its goal. The adaptive plans (i.e., 66 Gy to the initial PTV with a 12 Gy boost to the PTV after 50/60 Gy) and non-adaptive plans (78 Gy) were compared for 10 patients. Six patients obtained no benefit from the shrinking field method and the normal tissue constraints were still exceeded. Several issues, including the various methods used to define the threshold of treatment response between benign and malignant disease by PET, lung motion, prior treatment and sample size, may lead to variations. Moreover, we also speculate that the lack of selection by treatment response and the fact the mean volume decrease for the PTV was only 18-20% in these six patients may also explain these negative results. In patients with a small reduction in tumor volume, the benefits of the adaptive method are limited. In the present study, the mean reduction in the PTV for the shrinking field group was 38.6% (95% CI: 33.7-43.4%). A mean reduction of 26% (range: +15% to −75%) in CT-defined tumor volume was described by Feng et al. [13], with a residual GTV of 49% reported by Guckenberger et al. [9]. Another study [21] reported that if the GTV reduced by more than 30% at any point during the first 20 treatment fractions, adaptive planning was appropriate and could help to further improve the therapeutic ratio. This was the major reason why we only assessed good responders in this study.

We also considered local control and survival. The patients treated with adaptive plans received a higher dose of radiation, yet had larger initial GTV and PTV volumes. It is more challenging to devise a safe radiation plan for patients with large initial tumor volumes. Whether shrinkage of the radiation field results in higher incidences of locoregional progression or distant metastasis was explored in this study. Subclinical lesions are considered the leading cause of treatment failure. Compared with the non-shrinking field group, PFS significantly improved (14.0 months, 95% CI: 8.7–19.3 vs. 11.0 months, 95% CI: 9.3-12.7 months, *P* = 0.006) and the incidence of progression was significantly lower in the shrinking field group (95.7% vs. 66.0%, *P <* 0.001). These results suggest that the higher radiation dose delivered to the macroscopic tumor in patients with a large reduction in tumor volume may play a role in controlling disease progression. These observations are in agreement with Koay et al. [15], who found none of the nine patients who received adaptive replanning experienced local failure.

In this study, shrinking field radiation slightly reduced overall survival, though this difference was not statistically different. One reason that may partly explain this phenomenon is the larger tumor volume of the patients with tumor shrinkage may negatively impact prognosis [22, 23]. Moreover, different treatment after disease progression may influence OS. This was why we focused more attention on PFS than OS, as PFS may more accurately reflect the efficacy of shrinking field therapy. Thirdly, this retrospective cohort was small, therefore may be ofaffected by sampling bias.

This study has some limitations, including its retrospective design without random grouping and relatively small simple size, as mentioned above. Another potential drawback is the limited use of PET. Many studies [11, 12] have redelineated target volumes based on PET/CT, which can enable more accurate assessment tumor variations. Furthermore, spare radiation volume was not well proven by pathological analysis using modern diagnostic techniques in the current study. Histopathological confirmation is warranted for future studies of adaptive radiotherapy during chemoradiotherapy. This approach is currently being explored at our institute under a National Natural Science Foundation program (No. 81372438/H1610), via a phase II study comparing neoadjuvant chemoradiotherapy followed by surgery and definitive CRT after a good response to chemoradiotherapy, with adaptive radiation therapy guided by molecular imaging, in locally advanced NSCLC.

In conclusion, this study indicates that radiation field reductions for tumor shrinkage are feasible, acceptable and lead to a lower incidence of progression in locally advanced NSCLC. Additionally, the shrinking field group tended to have significantly improved median PFS and a lower incidence of ASIP. The adaptive method during chemoradiotherapy is a superior treatment for good responders, as it potentially spares normal tissues and enables dose escalation. Further clinical randomized controlled trials with a large sample size are warranted.

## MATERIALS AND METHODS

### Study population

Among the total of 381 consecutive patients who received radiotherapy between September 2009 and November 2014 in our institution, 217 received definitive chemoradiotherapy. Six patients stopped radiation midway due to personal choice. Among the remaining patients (who had varied responses to chemoradiotherapy), 97 patients with primary stage III NSCLC were evaluable in this study. The eligibility criteria were: (1) histologically- or cytologically-confirmed NSCLC; (2) a complete or partial response to chemoradiation; (3) no surgery due to unresectable tumors or the patient’s individual choice; (4) no other malignant neoplasms; (5) a Karnofsky Performance Status > 90 and no prior chemoradiotherapy; (6) and clinical stage III disease, according to the 7th edition of the American Joint Committee on Cancer. The patients who received shrinking field treatment were on a prospective clinical trial to investigate shrinking field therapy (Molecular imaging and molecular biomarkers based individual radiation therapy for non-small cell lung cancer) supported by Zhejiang Medicine & Health Key Research Fund (No.2012ZDA004), which was approved by the institutional review board of Zhejiang Cancer Hospital. This retrospective study was approved by the institutional review board, and signed informed consent was provided by every patient before starting therapy. The characteristics of the patients and tumors are summarized in Table 1.

### Treatment

All patients received concurrent or sequential chemoradiotherapy without surgery. Radiation oncologists delineated the target volume on multiple CT slices, with reference to PET/CT if necessary. The GTV was defined as the primary tumor and affected lymph nodes. The clinical target volume (CTV) was the GTV expanded by 6–8 mm, and the planning target volume (PTV) was generated by additional expansion not exceeding GTV 1.5 cm, considering microscopic tumor extension, mobility and daily setup errors. The structures of the normal lungs were outlined automatically and edited as appropriate to subtract the GTV, trachea and main bronchi. Radiation treatment plans were calculated using Pinnacle treatment planning system software (Philips Medical Systems, Milpitas, CA, USA). Tissue inhomogeneity corrections were applied to all plans. The total radiation dose to PTV was 50-70 Gy (median, 60 Gy) in conventional fractions (1.8-2 Gy per fraction, five fractions per week).

Computed tomography scans were performed after 40-50 Gy to evaluate the curative effect, leading to a boost in the radiation dose (6–20 Gy) with or without shrinking field radiation therapy using the same isocenter on the original mask, and the GTV and PTV were remeasured.

Platinum-based doublet chemotherapy was administered as first-line therapy. Platinum-based doublet chemotherapy was administered as first-line therapy. The regimens for squamous carcinoma were mainly taxanes, and pemetrexed for adenocarcinoma. The second-line therapy mainly depended on gene mutation status, histological type and site(s) of progression.

### Clinical evaluation and follow-up

Patients were evaluated as necessary during chemoradiotherapy, and routinely assessed every 3 months for 2 years, every 6 months for the next 3 years, and then annually. A clinical examination, blood tests, supraclavicular ultrasound, and thoracic and abdomen CT scans were included in each assessment. Brain MRI scans and bone ECT scans were obtained every year. Patients whose medical follow-up records were followed-up via telephone or mail.

Good responders were defined as patients with at least a 30% decrease in the sum of the diameters of target lesions (partial response) or disappearance of all target lesions (complete response) on the basis of the aforementioned examinations. Irradiation-induced lung injury was retrospectively scored using National Cancer Institute-Common Terminology Criteria for Adverse Events (CTCAE V4.0; for events occurring between day 1 and day 180 from the start of radiation treatment). A grade of 2 or above was defined as acute symptomatic irradiation-induced pneumonia (ASIP).

The date and site of progression (first failure) were determined through follow-up. Locoregional progression was defined as primary tumor recurrence or regional lymph node metastasis. Progression at any other site, including hematogenous metastases and development of malignant pleural or pericardial effusion, were considered evidence of distant progression. Progression-free survival (PFS) was calculated from the start of therapy to the first event (i.e., locoregional progression, distant metastasis, or death). Overall survival (OS) was defined as the time from the start of therapy to death. Follow-up data were last updated in January 2017. Three patients in the non-shrinking field group and one in the shrinking field group were lost to follow-up; only data on long-term survival outcome was missing for these patients.

### Statistical analysis

Statistical analysis was carried out using IBM SPSS 22.0 (IBM Corp., Armonk, NY, USA) according to the distribution of the data. The χ^2^ test or Fisher’s exact test were performed to compare proportions. The Mann-Whitney test was used to compare distributions of age and dose to the PTV. Normally-distributed variables were compared using Student’s *t*-tests. Results are presented with 95% confidence intervals (CIs). The Kaplan-Meier method was performed to assess PFS and OS in conjunction with the log-rank test. The four patients with missing survival outcome data were included in survival analysis. A two-tailed *P*-value of 0.05 or less was considered statistically significant.

## Abbreviations

NSCLC: Non-small Cell Lung Cancer
ASIP: Acute Symptomatic Irradiation-induce Pneumonia
3D-CRT: Three-dimensional Conformal Treatment
IMRT: Intensity-modulated Radiation Therapy
RILI: Radiation-induced Lung Injury
GTV: Gross Tumor Volume
PTV: Planning Target Volume
PFS: Progression-free Survival
OS: Overall Survival
